# Design and Fabrication of Wafer-Level Microlens Array with Moth-Eye Antireflective Nanostructures

**DOI:** 10.3390/nano9050747

**Published:** 2019-05-15

**Authors:** Shuping Xie, Xinjun Wan, Bo Yang, Wei Zhang, Xiaoxiao Wei, Songlin Zhuang

**Affiliations:** 1 Engineering Research Center of Optical Instrument and System, The Ministry of Education, University of Shanghai for Science and Technology, Shanghai 200093, China; 161390011@st.usst.edu.cn (S.X.); yangbo@usst.edu.cn (B.Y.); wei_zhang@usst.edu.cn (W.Z.); weixx@usst.edu.cn (X.W.); slzhuang@yahoo.com (S.Z.); 2 Ministry of Education and Shanghai Key Laboratory of Modern Optical System, University of Shanghai for Science and Technology, Shanghai 200093, China

**Keywords:** wafer-level microlens array, multiscale functional structures, moth-eye antireflection nanostructures, aspherical microlenses, nanoimprint lithography

## Abstract

Wafer-level packaging (WLP) based camera module production has attracted widespread industrial interest because it offers high production efficiency and compact modules. However, suppressing the surface Fresnel reflection losses is challenging for wafer-level microlens arrays. Traditional dielectric antireflection (AR) coatings can cause wafer warpage and coating fractures during wafer lens coating and reflow. In this paper, we present the fabrication of a multiscale functional structure-based wafer-level lens array incorporating moth-eye nanostructures for AR effects, hundred-micrometer-level aspherical lenses for camera imaging, and a wafer-level substrate for wafer assembly. The proposed fabrication process includes manufacturing a wafer lens array metal mold using ultraprecise machining, chemically generating a nanopore array layer, and replicating the multiscale wafer lens array using ultraviolet nanoimprint lithography. A 50-mm-diameter wafer lens array is fabricated containing 437 accurate aspherical microlenses with diameters of 1.0 mm; each lens surface possesses nanostructures with an average period of ~120 nm. The microlens quality is sufficient for imaging in terms of profile accuracy and roughness. Compared to lenses without AR nanostructures, the transmittance of the fabricated multiscale lens is increased by ~3% under wavelengths of 400–750 nm. This research provides a foundation for the high-throughput and low-cost industrial application of wafer-level arrays with AR nanostructures.

## 1. Introduction

The production of micro camera modules for mobile phones and other Internet of Things (IOT) terminals has developed into a large market. Although micro camera module production is mainly accomplished by the vertical assembly of discrete lenses, the wafer-level packaging (WLP) approach for micro cameras has attracted widespread attention because it offers compact module sizes and high production efficiency [1]. In the WLP process, several wafer-level dense microlens array plates are vertically assembled after wafer-scale registration, and then cut into thousands of discrete micro camera modules. The resulting camera module is a compact cube free of the traditional lens housing components. The wafer-level dense microlens array is the core component for WLP production. However, suppressing the ~4% Fresnel reflection of wafer-level microlens arrays to <1% remains a major challenge. The traditional approach uses antireflection (AR) coatings deposited on the surfaces of the lenses, such as quarter-wavelength multilayer dielectric coatings [2,3,4,5]. However, the wafer-level microlens array of 4–8 inches in diameter has a low thickness of several hundred micrometers, which yields a high probability of wafer warpage during coating deposition. Wafer warpage can seriously damage wafer registration. Moreover, micro camera modules must withstand high-temperature reflow processes during the process of welding onto circuit boards. Dielectric AR coatings can rarely survive such processes, because their thermal expansion coefficient mismatches with the base microlens material, and can cause coating fracture [6,7,8,9]. These factors indicate that wafer-level microlens array production requires new AR solutions.

Nature offers a possible answer to this problem. Moth-eye biomimetic nanostructures, first found by Bernhard [10], show great promise in reducing surface Fresnel reflections and enhancing transmission over broad wavelength bands [11,12,13,14]. Numerous studies have verified the AR effect of moth-eye structures and applied them to improve the efficiency of photovoltaic devices, light emitting diode (LED) coupling, and liquid crystal display (LCD) displays [15,16,17,18]. Previous studies have mainly simulated, fabricated, and tested moth-eye nanostructures on planar substrates or flexible material-based samples [3,7,8,9,13,14,15,16]. For fabrication, interference lithography is usually applied to generate the required 2D periodic pattern on a photoresist layer, followed by etching to transfer the pattern to a substrate and form the moth-eye surface [19,20]. Lee et al. [21] studied the superhydrophobicity of nanostructures and microstructures fabricated by lithography and Al etching on polymer surfaces. The etched substrate can also serve as a mold for nanoimprinting, which can produce large-area nanostructured surfaces with a roll-to-roll setup. However, such a process cannot be easily transferred to the production of dense wafer-level microlens arrays. Firstly, the periodically curved surface of the microlens array impedes the uniform deposition of photoresist by spin-coating. Lithography on curved surfaces is also hard to control. Moreover, any potential moth-eye nanostructure fabrication process must easily fit into the current wafer lens array production process to enable adoption in industrial manufacturing. In a previous report, moth-eye nanostructures were fabricated on a microlens array, but the microlenses had to be thermally imprinted, which is unsuitable for WLP processing [6]. Thus, few reports exist regarding the application of moth-eye AR structures in WLP production.

This study proposes a process for fabricating moth-eye nanostructures on wafer-level microlens arrays, and reports for the first time, a multiscale functional structure-based precision optical component encompassing hundred-nanometer-level nanostructured arrays for AR effects, hundred-micrometer-level microlens arrays for camera imaging, and a centimeter-level wafer substrate for the WLP process flow. The nanostructures here not only satisfy the AR requirement of the microlens array, but also avoid coating-fracture during thermal reflow. In order to achieve high-throughput and cost-effective production, a high-precision metal mold for wafer lens arrays with moth-eye nanostructures is fabricated based on a diamond milling process and subsequent anodic aluminum oxidation (AAO) treatment. The mold, and an ultraviolet (UV) nanoimprint lithography process are then used to fabricate a wafer bearing a microlens array with AR nanostructures. To verify the feasibility, a 50 mm wafer sample containing 437 aspherical microlenses is fabricated. Each 1-mm-diameter lens has an evenly distributed array of nanometer structures with an average period of ~120 nm. The wafer and microlens quality satisfy the WLP requirements. Compared to a lens array fabricated without AR nanostructures, the transmittance is increased by ~3% for wavelengths between 400 nm and 850 nm.

The proposed method is suitable for current WLP processing because it only modifies the metal mold to include moth-eye nanostructures. This research thus provides a foundation for the industrial adoption of wafer-level microlens arrays with moth-eye nanostructures.

This article is structured as follows: Section 2 introduces the theory and simulation of the moth-eye AR structure, Section 3 describes the fabrication process design and experimental results, and Section 4 presents the fabricated sample analysis and discussions.

## 2. Moth-Eye AR Theory and Simulation

**Figure 1:**
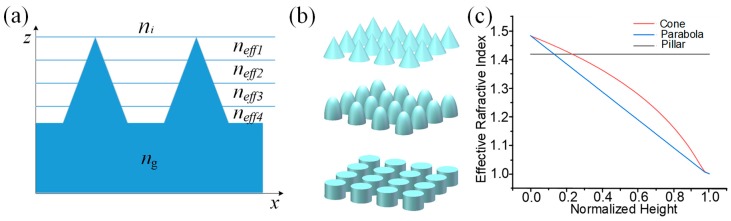
(**a**) Schematic of the effective refractive index gradient of moth-eye nanostructured surface. (**b**) Nanostructure shapes: Cone-shaped array, parabola-shaped array, pillar-shaped array; (**c**) change of effective refractive index as incident light travels from air (*n_i_* = 1.0) into the substrate (*n_g_* = 1.53). Normalized height of 1 represents the peak of the AR (antireflection) nanostructures, that of 0 indicates the base substrate.

The moth-eye nanostructured surface is characterized by a uniform layer of quasi-periodical protuberances on a substrate [22]. The lateral period of the nanostructures is much smaller than the optical wavelengths and is thus equivalent to a diffraction grating where only the zeroth order can propagate, while all other orders are evanescent [23,24]. The moth-eye layer is optically equivalent to a film with a refractive index gradient in its depth direction [25], rather than the abrupt change in index that occurs in films without nanostructures. Figure 1a shows schematically the continuous increase of the physical thickness of the nanostructures along the AR structured surface from air to the bulk. The moth-eye nanostructure can thus be modeled as a multilayer system with different effective refractive indices [26,27,28,29,30].

The effective refractive index *n_eff_* for normal incidence at each layer can be written as:

(1) $${n_{eff}}^{2}=f\times {n_{g}}^{2}+\left(1-f\right){n_{i}}^{2}$$

where *n_i_* is the refractive index of air, *n_g_* is the refractive index of the substrate, and *f* is the fill factor of the periodic structure [26,28]. According to Equation (1), for the same wavelength and material, *f* affects the effective refractive index. Figure 1b shows three common nanostructure shapes, and Figure 1c exhibits the variation of the effective refractive index with the three differently shaped nanostructures. For the pillar-shaped array, its *f* is constant from top to bottom; therefore, its effective refractive index is constant, like that of a single-layer coating. The pillar-shaped nanostructure thus cannot achieve good AR properties over a broad spectral range. Cone- and parabola-shaped nanostructures generate smoother transitions in refractive index from the air to the substrate bulk, implying better AR effects.

**Figure 2:**
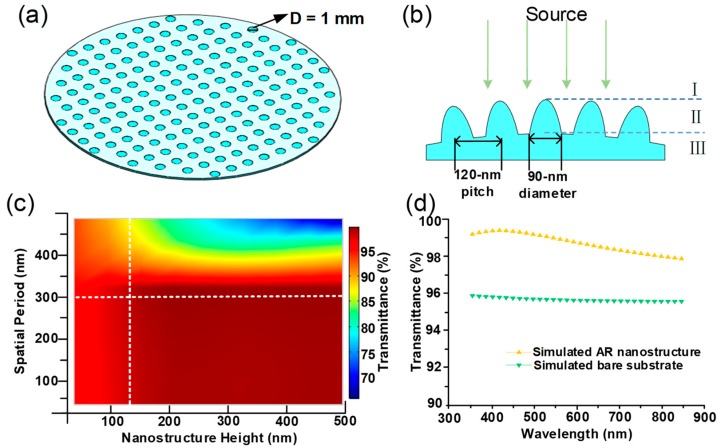
Simulation model: (**a**) Wafer-level microlens array; (**b**) parabola-shaped nanostructures over a lenslet surface; (**c**) simulated optical transmission of parabola-shaped nanostructures with different spatial periods and heights under 550 nm irradiation; (**d**) simulated transmission spectra of bare substrate, and of parabola-shaped array with the bottom diameter = 90 nm, period = 120 nm, and height = 150 nm.

Based on the above analysis, the parabola-shaped nanostructure array was selected for further detailed simulation. The finite-difference time-domain (FDTD) simulation was used to determine the optimum parameters of the AR nanostructure in the spectral range of 350–850 nm. The geometries of the wafer lens array and AR nanostructures on each microlens are illustrated in Figure 2a,b, respectively. The nanostructured region II separates regions I (air) and III (lens material); *f* at the substrate is set to 0.75. The calculated transmittance at a center wavelength of 550 nm for different nanostructure settings is presented in Figure 2c. When the spatial period is much smaller than the incident wavelength, the transmission is not greatly affected by variations in the period. However, as the spatial period approaches the incident wavelength in size, first-order diffraction begins to occur, causing a significant decrease in transmission. The height of the AR nanostructures also affects the transmission. Figure 2c shows that within the simulated height range, the transmission is proportional to the height for the same period. Figure 2d shows the simulated transmission spectrum of a surface with the nanostructure period of 120 nm and height of 150 nm, which are similar to the nanostructure parameters of the fabricated sample produced later. The simulated AR effect is adequate for WLP applications.

## 3. Fabrication Process

### 3.1. General Process Flow

After the optimization of the moth-eye nanostructure, a cost-effective and high-output process for its fabrication on dense wafer-level lens arrays must be developed for commercial application. Different techniques such as e-beam writing [31,32], mask lithography [33], and interference lithography [34,35] have been applied to fabricate moth-eye nanostructures. These methods fail to accommodate the large size and curved surfaces of wafer lens arrays, as mentioned above. The method proposed here combines a multiscale structure-based metal mold and UV nanoimprint lithography to fabricate moth-eye nanostructures on the wafer-level microlens array.

**Figure 3:**
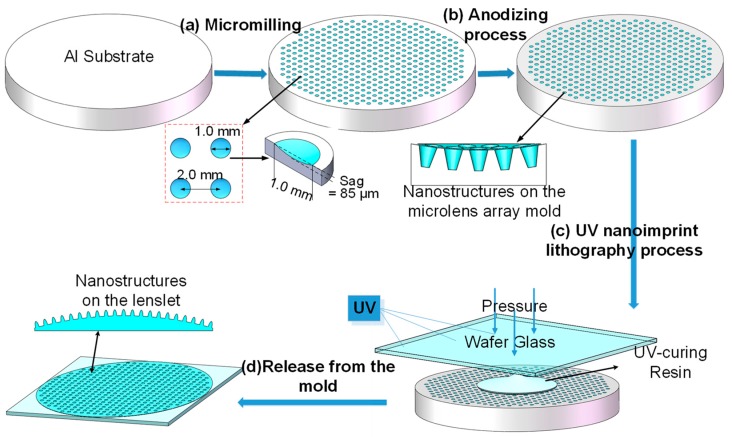
Schematic of the fabrication process for a wafer-level microlens array with antireflection (AR) nanostructures.

The process for producing AR nanostructures on the wafer-level lens array is depicted in Figure 3. Firstly, a 4–8-inch-diameter (10–20.3-cm-diameter) aluminum plate, with the diameter matching the targeted wafer lens array size, was manufactured into a high-precision wafer-level lens array mold using a diamond milling machine according to the design of the microlens surface. The microlens surface could be spherical, aspheric, or free-form in shape [36,37,38]. Secondly, the metal mold was treated using a chemical approach to create an even layer of V-shaped nanopores on the hard mold [39]. Next, a UV-curable resin-based nanoimprint process was used to transfer the hybrid structures on the metal mold to a glass wafer substrate, generating the wafer lens array with nanostructured surfaces. Finally, the wafer-level lens array was released from the mold after curing. To achieve a concave microlens array, the above wafer lens array could serve as an intermediate mold; another nanoimprinting process with steps similar to the one mentioned above could be performed to deposit the array on a curved substrate. This process matches well with the current, mainstream wafer lens array manufacturing process, and therefore, has a high probability of industrial applicability.

To verify the feasibility of the process, a 50-mm-diameter wafer lens array with 437 aspherical lenslets with cavity apertures of precisely 1.0 mm, patterned with 120-nm-pitch parabola-shaped nanostructures was manufactured following the above process flow.

### 3.2. Fabrication of the Precision Hybrid-Structured Metal Wafer Mold

**Figure 4:**
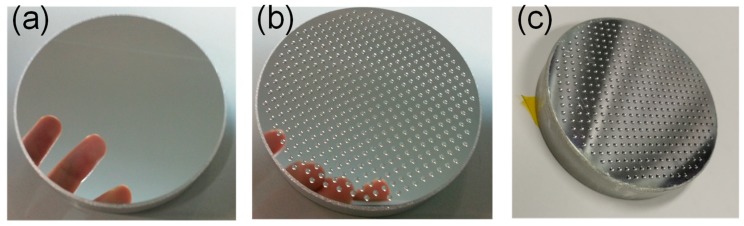
Different stages in the fabrication of the metal wafer mold for the hybrid-structure microlens array: (**a**) Diamond turning yields a mirror-flat substrate; (**b**) micro-milled microlens mold cavities on the substrate; (**c**) the obtained wafer mold after chemical generation of a uniform layer of nanopores on the surface.

The fabrication of the wafer-level lens array mold with hybrid structures was the most critical step of the process. Wafer-level microlens arrays have very high dimensional accuracy requirements for the profiles and positioning of the lens. To meet these requirements, the dense microlens array mold was fabricated on an ultraprecise machine (350FG, Moore Nanotechnology, Swanzey, NH, USA) with five-axis servo motion. After numerous trials to identify the right material, such as single-crystal versus polycrystalline pure aluminum, and different aluminum alloys, an aluminum alloy (6061) substrate electroplated with ~0.3-mm-thick pure aluminum comprising nanosized grains (AlumiPlate, Coon Rapids, MN, USA) was finally selected as the mold material. This was done by considering the requirements of both the ultraprecise machining process and the subsequent chemical AAO process. A diamond turning process was used to create an optically flat surface on the 50-mm-diameter aluminum substrate, as shown in Figure 4a. Next, a concave and dense microlens array was machined by a micro-milling process using a high-speed air-bearing spindle, as shown in Figure 4b. The resulting wafer mold contained 437 accurate aspherical lens mold cavities with a cavity aperture of precisely 1.0 mm and a sag of 85 μm. The surface roughness (*S*_a_) of the resulting mold was ~4 nm.

**Figure 5:**
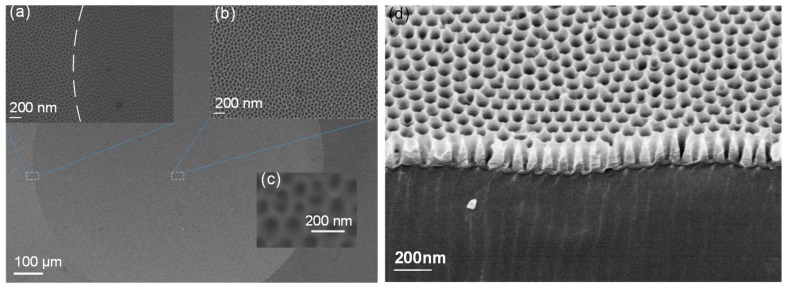
The scanning electron microscope (SEM) image of the nanostructured microlens mold: (**a**) Magnified SEM image of the lenslet boundary region, where the boundary is indicated by the dashed line; (**b**) magnified SEM image of the lenslet apex region; (**c**) a detailed view of the nanopore structure; (**d**) a Cross-sectional image of the nanopores (height of nanopores ~150 nm).

In order to generate a uniform layer of nanostructures on the lens array mold, we adopted the AAO process, which has been widely examined on aluminum sheet samples for nanopore templating applications [40,41,42]. However, the suitability of this chemical process for curved array surfaces, and its capacity to maintain the surface accuracy and minimal roughness of a mirror-quality precision mold was unknown. The extruded pure aluminum sheet material commonly used for AAO was unsuitable for wafer mold ultraprecise machining. In these experiments, we placed the machined dense microlens array mold in an oxalic acid electrolyte, and investigated its characteristics, such as pore size, interpore distance, and nanolayer thickness by varying the applied voltage, electrolyte concentration, and reaction time. In the first anodization step, the clean microlens array mold was anodized in the oxalic acid solution at room temperature (25 °C), at a voltage of ~40–50 V; a mixed solution of H_3_PO_4_ and H_2_C_r_O_4_ was used to remove the oxidized layer. Then, a pore-widening process was performed using H_3_PO_4_ for ~30 min. After parametric tuning of this two-step anodization process, a nanostructured mold was obtained with an average pore diameter of ~100 nm and spatial period of ~120 nm, as shown in Figure 4c. The mold nanostructure was characterized using a ZEISS scanning electron microscope (SEM; Carl Zeiss Jena, Oberkochen, Germany), as shown in Figure 5. The SEM image shows the uniformity of the nanopore layer on the microlens mold cavity, both at the apex flat region, and the lens boundary region. The cross-sectional SEM images of the thick metal mold were difficult to record, so Figure 5d shows a SEM image of a nanostructured sheet plate sample-fabricated using the same process parameters as those of the metal mold. The nanopores were approximately V-shaped. Because the mold was very delicate, further examination was performed on the final replicated lens array sample.

### 3.3. UV Resin-Based Nanoimprint Lithography Process

**Figure 6:**
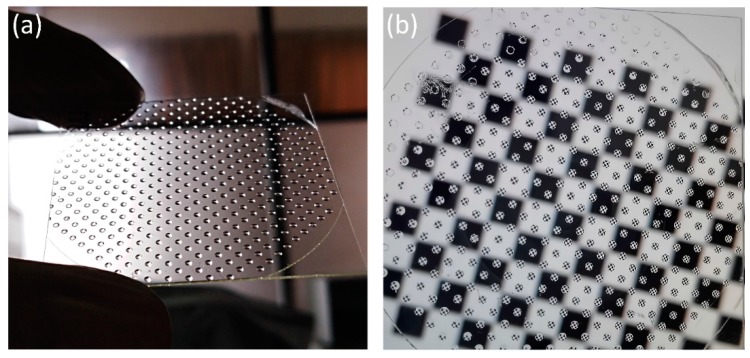
Photographs of wafer-level microlens array with AR nanostructures: (**a**) Image of the fabricated microlens array; (**b**) view of a checkerboard pattern placed at the focal point of the microlens array.

UV nanoimprint lithography was then adopted to transfer the hybrid structures on the metal mold fabricated above to a glass substrate. Firstly, the metal mold was cleaned with water and acetone, dried at >100 °C, and then coated with a silicone oil demolding layer (Dow Corning OS-20, Krayden, Inc., Denver, CO, USA). Secondly, the mold was placed in a vacuum chamber for ~5 min, while the UV-curable liquid resin (Ormocomp, Micro Resist, Berlin, Germany) was dispensed over the metal mold. Next, a clean glass wafer was placed over the mold and pressed slowly into the resin. The mold was then placed on a heating oven, and heated for ~10 min at ~100 °C. UV light was then used to cure the resin at an exposure intensity of ~20 mW/cm^2^ for 5 min. Finally, the replicated microlens array with AR nanostructures on the glass substrate was mechanically detached. Figure 6 shows the resulting wafer lens array with micro- and nanoscale hybrid structures. The fabricated sample is only 50 mm in diameter, but the process can be adapted to larger-format wafer lens fabrication by using a larger metal mold.

## 4. Sample Analysis and Discussion

**Figure 7:**
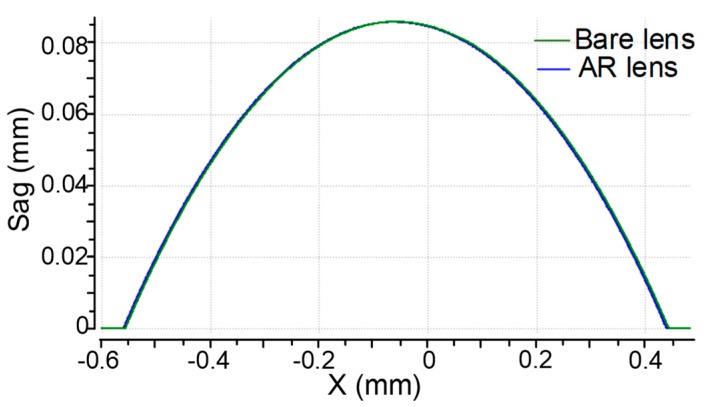
Profile stylus measurement results for lenslets with and without AR nanostructures.

**Figure 8:**
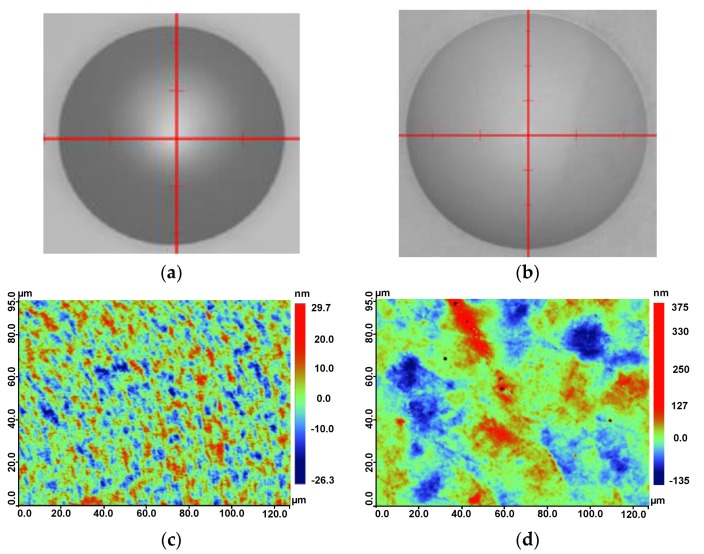
The microscopic appearance of lenslets: (**a**) without AR nanostructures; (**b**) with AR nanostructures; (**c**) surface roughness of lenslets without nanostructures (*S*_a_ = 5.2 nm); (**d**) surface roughness of lenslets with nanostructures (*S*_a_ = 15.5 nm).

The obtained multiscale structured wafer lens array was examined to study its surface profile accuracy, surface roughness, nanostructure quality, and AR performance. The analyses results were compared to those from a common wafer lens array without nanostructures for reference. This array was replicated from the same metal mold before AAO treatment. The profiles of the two microlens array samples were measured with a stylus profiler phase grating interferometer (PGI) Dimension (Taylor Hobson, Leicester, UK). Figure 7 demonstrates the profiles of the microlens arrays with and without AR nanostructures. The two profiles were overlaid for easy comparison. The apertures of both the lenses were 0.996 mm with a sag of ~84.5 μm, nearing design values of 1.0 mm and 85 μm. The good conformity of the profiles illustrated that the geometry of the AR-structured mold was replicated accurately, which was critical to the imaging performance of the final lens. The surface roughness was another important quality factor. Figure 8 exhibits the appearance and roughness of the samples, measured using a white light interference microscope (Contour GTK, Bruker, Karlsruhe, Germany). The AR nanostructured surface roughness was ~15.5 nm, which was still of mirror quality, but larger than the surface roughness of the bare lens (*S*_a_ = 5.2 nm). The *S*_a_ value increase was largely attributed to the presence of nanostructures compared to the smooth surface. Another possible source of roughness could be the AAO chemical reaction, which may have modified the surface micro-topology of the mold as shown in the measured color-rendered height maps. Visual inspection indicated no obvious scratches or other defects. The results of the profile and surface roughness measurements showed that the replicated AR nanostructured lens could meet the requirements for precision optical imaging.

**Figure 9:**
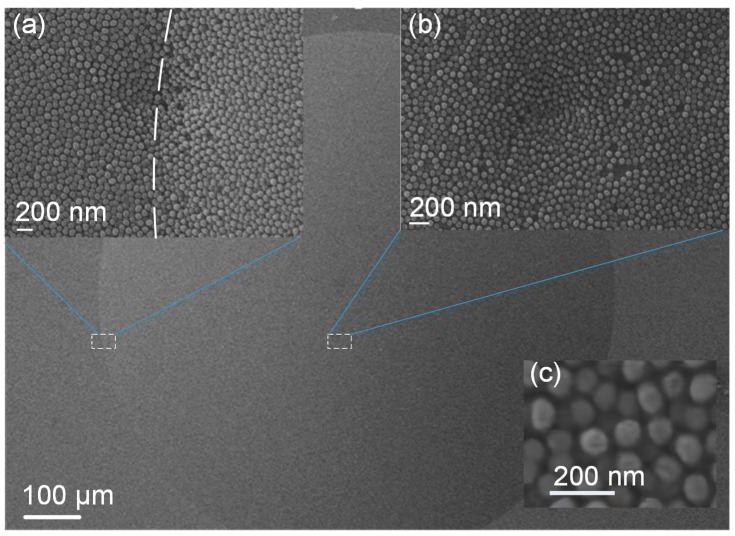
The SEM image of the replicated microlens with AR nanostructures: (**a**) Magnified SEM image of the lenslet boundary region. The dashed line is the lenslet boundary; (**b**) magnified SEM image of the lenslet apex region; (**c**) a detailed view of the AR nanostructures.

**Figure 10:**
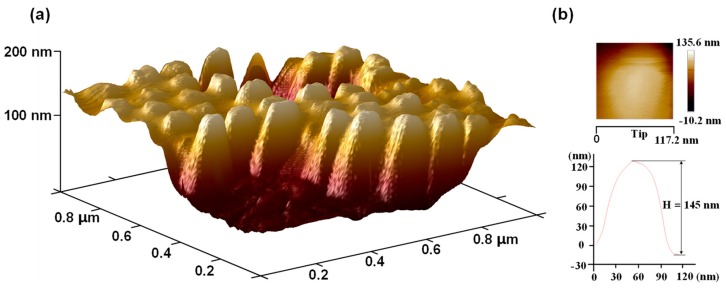
Atomic force microscopy (AFM) characterization of the AR nanostructures replicated from the nanostructured microlens mold: (**a**) Three-dimensional AFM image over area of 1 μm × 1 μm; (**b**) tip estimation and line profile of height using AFM.

The SEM images of the fabricated AR nanostructured microlens array sample are shown in Figure 9. An even layer of nanostructures is distributed on the lens surface, as expected. The magnified views (Figure 9a,b) of the apex and the boundary regions demonstrates that the nanostructures have equal parameters and qualities. In Figure 9, the SEM images both exhibit local surface-sinking phenomena in the center of the images, which arises from damage to the polymer caused by the focused electron beam during the SEM scan process. The three-dimensional structural parameters of the AR nanostructures were obtained using atomic force microscopy (AFM; Multimode8, Bruker, Karlsruhe, Germany). Figure 10a presents the AFM images of the AR parabola-shaped nanostructures over an area of 1 μm × 1 μm. As shown in Figure 10b, the tip estimation and height characterization of the nanostructure are measured from the AFM image. The height and period of the nanostructures were 145 nm and 117.2 nm, respectively, which are close to the simulated parameters of 150 nm and 120 nm. It is reasonable that the shape of the fabricated nanostructures is similar to a parabola. Therefore, we compared the measured AR performance of the fabricated nanostructures with the simulation of parabola-shaped nanostructures in the subsequent analysis.

**Figure 11:**
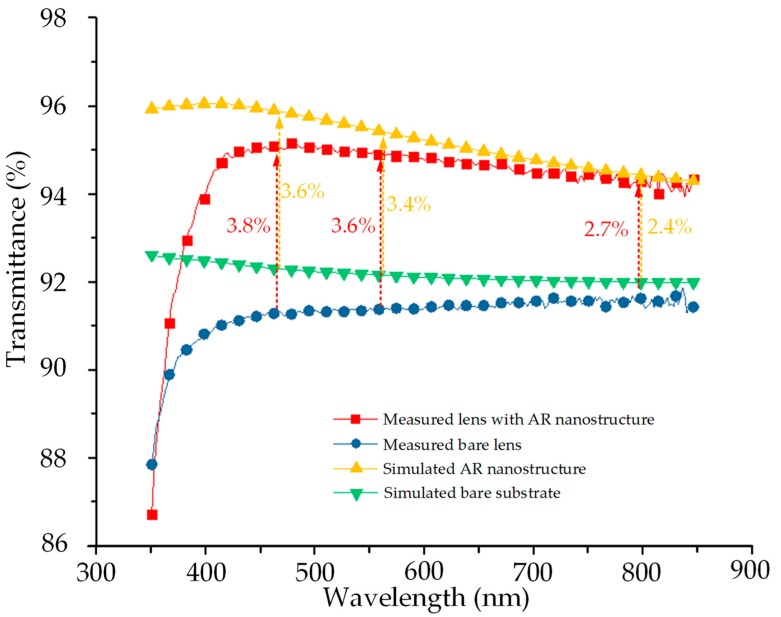
Experimental and simulated optical transmittance spectra for the replicated microlens arrays with AR structures and the bare microlens array under wavelengths from 350–850 nm. The experimental data clearly show 3.8%, 3.6%, and 2.7% improvements (red arrows) at the wavelengths of 450, 550, and 800 nm, respectively. The experimental results agree well with the simulated results (yellow arrows).

Spectrophotometry measurements (Lambda 1050, PerkinElmer, Waltham, USA) were performed to measure the transmittance spectra of both samples under normal incidence. The results are presented in Figure 11. For each measurement, a single lens area was used to calculate the transmittance test. As seen in Figure 11, the transmittance of the AR nanostructured lens was increased by >3% over the visible spectral bandwidth, compared to that of the bare lens. The AR performance agreed well with the simulation result in both the trend and magnitude of the transmittance improvement. Therefore, the AR performance of the moth-eye nanostructures was equivalent to that of a dielectric AR coating, but the moth-eye coating avoided the risk of wafer warpage and coating fracture.

## 5. Summary

We demonstrated the fabrication of a high-quality multiscale structure-based wafer-level microlens array, which included hundreds of accurate aspherical microlenses and a uniform layer of moth-eye AR nanostructures on each single lens. The moth-eye AR design avoids the issues of wafer warpage and coating fracture in WLP processing. The proposed fabrication process is compatible with current mainstream wafer-level production processes, using a nanostructured metal wafer mold and UV nanoimprinting. The feasibility of the process is demonstrated by the fabrication of a 50-mm-diameter wafer lens array with AR nanostructures. The quality of both the microlens and the nanostructures approaches the levels required for real applications. The moth-eye AR nanostructures on the microlens can achieve >3% transmittance increase for a single interface throughout the visible wavelength range, as verified both in simulations and real spectrometry tests.

There is more room for further research. The real aspherical lens sag in production may be much larger than was observed in the current tests, and this would correspond to larger surface slope angles. For large slope angles, the angle-dependence of the moth-eye nanostructure AR effect will begin to manifest, and there is also a greater risk of mold detachment failure during UV imprinting. Scaling the proposed process to a 6–8-inch wafer lens array production also faces technological uncertainties. Considering these issues, the study here still establishes a good foundation for the future research and adoption of moth-eye AR nanostructures in the WLP industry.
